# Neonatal bacterial meningitis in Tikur Anbessa Specialized Hospital, Ethiopia: a 10-year retrospective review

**DOI:** 10.1186/s40064-016-3668-1

**Published:** 2016-11-14

**Authors:** Melese Abate Reta, Tamrat Abebe Zeleke

**Affiliations:** 1 Department of Medical Laboratory Science, Faculty of Health Science, Woldia University, P.O. Box 400, Woldia, Ethiopia; 2 Department of Microbiology, Immunology and Parasitology, School of Medicine, Addis Ababa University, Addis Ababa, Ethiopia

**Keywords:** Neonates, Bacterial meningitis, *Streptococcus pneumonia*, *E. coli*

## Abstract

**Background:**

Bacterial meningitis is still a major public health threat in developing countries. It is an overwhelming infection with a high morbidity and mortality rate, especially in neonates. The aim of this study was to determine the prevalence and etiological agents that cause bacterial neonatal meningitis at Tikur Anbessa Specialized Hospital (TASH).

**Methods:**

This is a retrospective analysis of 1189 cerebrospinal fluid (CSF) specimens submitted to the bacteriology laboratory of TASH for culture from 2001 to 2010. All newborns younger than 29 days old that were suspected for bacterial meningitis cases were included in the study.

**Results:**

Based on CSF culture, 56 newborns were identified as having bacterial meningitis from a total of 1189 suspected cases. The overall prevalence of neonatal bacterial meningitis from the total suspected cases was 4.7%. The organisms identified and their prevalence rates were *Streptococcus pneumoniae* 13 (23%), *Escherichia coli* 9 (16%), *Acinetobacter* 7 (13%), *Neisseria meningitides* 5 (9%), *Klebsiella* spp. 5 (9%), *Staphylococcus aureus* 3 (5%) and *Streptococcus pyogen* 3 (5%). There were two (4%) cases each that was caused by *Coagulase*-*Negative*-*Staphylococcus* and *Non*-*Group*-*A*-*Streptococcus*, while 1 (2%) caused by *Haemophilus influenzae*. *S. pneumoniae* was the main etiological agent identified from CSF culture. The male to female ratio was 1:0.88 (53% were male). The birth weights of 34 (61%) patients were under 2500 g, and 22 (39%) patients had normal birth weights. Twenty-seven (48%) were early onset cases, and 29 (52%) were late-onset.

**Conclusion:**

*Streptococcus pneumoniae* and *Escherichia coli* were the two main etiological agents for neonatal bacterial meningitis infection in the study area.

## Background

Acute bacterial meningitis occurs more commonly during the first month of life than any other subsequent period and it is associated with high morbidity and mortality. Neonatal meningitis is an illness characterized as a result of infection of the meninges and it typically happens between birth and the first 28 days of life (Mohammad et al. 2010; Luzia et al. 2007; Delouvois et al. 1991). Neonatal meningitis is an important cause of morbidity in sub-Saharan Africa and requires urgent empiric treatment with parenteral administered antibiotics (Olivia et al. 2014).

The incidence of neonatal meningitis in western countries varies from 0.2 to 0.5 cases per 1000 live births but much higher rates of 1.1–1.9 per 1000 have been reported from developing countries (Laving et al. 2003). The mortality varies based on the treatment, with survival rates of 17–29% and with complications rates of 15–68% (Luzia et al. 2007). Two-thirds of meningitis deaths in low income countries occur among children under 15 years of age (Meenakshi et al. 2009).

The causative agents of neonatal bacterial meningitis are different geographically. In most developed countries the main causative agents for neonatal bacterial meningitis isolated from cerebrospinal fluid (CSF) are *Group B Streptococcus* (*GBS*), *E. coli*, *Listeria monocytogenes* and *S. pneumonia* (Harvey et al. 1999; Delouvois et al. 1991; Pong and Bradley 1999). Infections in the neonatal period are ‘early onset’ (implying vertical transmission) when frequently isolated bacteria include *GBS*, *E. coli* and *L. monocytogenes*, and ‘late onset’ (implying nosocomial or community acquired infection). The microorganism spectrum responsible for neonatal meningitis in developing countries is different. The Reasons for this may include genetic differences in immune response and possibly geographic differences in laboratory techniques for pathogen isolation and reporting and they are also influenced by maternal and infant risk factors, and prevention and treatment strategies of the country (Bozena et al. 2005; Osrin et al. 2004; Heath et al. 2003). The bacteria causing neonatal meningitis not only vary between different countries, but also show temporal changes within the same country (Mohammad et al. 2010). In developed countries infection with gram-negative bacilli accounts for 30–40% of meningitis case (Dawson et al. 1999). The disease is often more severe with gram-negative bacteria than with gram-positive bacteria with higher rates of both mortality and morbidity. Neonatal and maternal risk factors for developing neonatal meningitis include low birth weight, prematurity, premature ruptures of membranes, maternal chorioamnionitis and low socioeconomic status (Polin and Harris 2001; Osrin et al. 2004). The newborn that is particularly susceptible to infection as the immature immune system is deficient in humoral and cellular immune responses (Saleem et al. 2014; Pablo et al. 2007).

Signs of meningitis are often subtle in the neonate; thus, meningitis must be diagnosed by examination of CSF. Culturing the CSF is a proven test for demonstration of bacterial meningitis (Mohammad et al. 2010). Other tests, including the evaluation of glucose, protein, and leukocyte levels in the CSF can help to diagnose bacterial meningitis. However, Harmony et al. (2006) have stated that neonates with bacterial meningitis cannot be diagnosed accurately with CSF glucose or protein evaluation because of lack of specificity due to a wide distribution of normal values in this age group (Matthijs et al. 2010). Our study was carried out by identifying etiological agents and assessing the association between gestational age, birth weight, onset type and bacterial meningitis.

## Methods

### Study population

The study was carried out at the Tikur Anbessa Specialized Hospital (TASH), Ethiopia. The records of all neonates admitted to the general wards of the hospital from 2001 to 2010 were retrieved from the records department. All neonates suspected with bacterial meningitis (BM) infection cases were first identified from the bacteriology laboratory registers. The neonatal period was defined as that time from birth to 28 days of age. A pre-coded data sheet was used to extract information from each record. The patient was identified by hospital inpatient number and study number given in consecutive sequence. The following data were abstracted from the charts: Gender, birth weight, gestational age, type of infections and isolated organism. The data were analyzed on the basis of gender, type of infection [early-onset infection (≤7 days), late-onset infection (>7 days) and gestational age of the neonates]. Infants were categorized according to their birth weight into three groups: Normal weight (≥2500 g), low birth weight (LBW) (1500–2499 g) and very low birth weight (VLBW) (1000–1499 g). Gestational age was recorded as term (≥37 weeks) or preterm (<37 weeks), and the information on mean of bodyweight, mean of gestational age and isolation of bacterial agents was recorded.

### Laboratory methods

Approximately 0.5–1.0 ml of CSF obtained aseptically via a lumbar puncture was collected in two specimen bottles and taken immediately to department of bacteriology laboratory, TASH. The sediment of centrifuged CSF from the sterile bottle was inoculated using a sterile loop onto chocolate, blood agar and MacConkey’s agar plates (Hi-Media, Mumbai, India) and incubated at 35–37 °C overnight in candle extinction jars to provide 5–8% carbon dioxide. The plates read every 24 h for 3 days. In case of a growth, the isolates would be processed and identified by standard bacteriological techniques. Cases were defined as meningitis if the CSF culture was positive for aerobic bacteria.

### Ethical considerations

This being a retrospective study, ethical considerations was fulfilled by obtaining permission from the ethics and research committee of the Hospital. Thereafter inpatient registration number was used to identify the participants and not names; hence, this ensures confidentiality.

## Results

### Characteristics of study population

During the 10 year period considering this retrospective study 1189 neonate patients with suspected bacterial meningitis were examined using CSF culture in bacteriology laboratory at TASH. Six hundred thirty-two (53%) were male, while 557 (47%) were female; making male to female ratio of 1:0.88. The results of this study indicated that 533 (45%) suspected cases of neonatal bacterial meningitis were late onset and 656 (55%) were early onset. Three hundred fourteen of the 533 early-onset cases and 352 of the 656 late-onset cases weighed under 2500 g. The mean birth weight of the neonates with BM suspected case was 2229 g (range: 1120–4100 g), of which 422 (35%) had normal birth weight, 614 (52%) had LBW and 153 (13%) had VLBW. According to the gestational age, 601 (51%) suspected cases were preterm and 588 (49%) cases were term (Table 1).

**Table 1:** Gender, types of infection, gestational age and birth weight of neonates diagnosed with neonatal bacterial meningitis suspected cases

Variables	Frequency	Percent
Gender		
Male	632	53
Female	557	47
Total	1189	100
Type of infection		
Early onset	533	45
Late onset	656	55
Total	1189	100
Gestational age of neonates		
Preterm	601	51
Term	588	49
Total	1189	100
Birth weight of neonates		
≥2500 g	523	44
<2500 g	666	56
Total	1189	100

### Bacterial culture data

Of the total 56 positive cases, the bacteria identified and their prevalence rates were *S. pneumoniae* 13 (23%), *E. coli* 9 (16%), *Acinetobacter* 7 (13%), *N. meningitidis* 5 (9%), *Klebsiella* spp. 5 (9%), *S. aureus* 3 (5%) and *S. pyogen* 3 (5%). There were two (4%) cases each that was caused by *Coag. Negative*-*Staphylococcus*, *Non*-*Group*-*A*-*Streptococcus*; while 1 (2%) were caused by *H. influenzae*. The main etiological agent identified from CSF culture was *S. pneumoniae*. Nine percent of *S. pneumoniae* cases were isolated from LBW and VLBW neonates. The major causative agents for neonatal meningitis cases identified were isolated from newborns with LBW and VLBW (61%) (Table 2).

**Table 2:** Frequency of isolated bacteria in neonates with bacterial meningitis according to birth weight, onset type, and gestational age

Organisms found in CSF culture	Birth weight		Onset type		Gestational age	
	LBW <2500 g n (%)	NBW ≥2500 g n (%)	Early n (%)	Late n (%)	Preterm n (%)	Term n (%)
No bacterial growth	632 (95)	501 (96)	506 (95)	627 (96)	578 (96)	555 (94)
*Klebsiella* spp.	3 (0.5)	2 (0.4)	3 (0.6)	2 (0.3)	3 (0.5)	2 (0.3)
*S. pneumoniae*	5 (0.8)	8 (1.5)	6 (1.1)	7 (1.1)	4 (0.7)	9 (1.5)
*S. pyogen*	1 (0.2)	2 (0.4)	1 (0.2)	2 (0.3)	1 (0.2)	2 (0.3)
*S. aureus*	2 (0.3)	1 (0.2)	2 (0.4)	1 (0.2)	1 (0.2)	2 (0.3)
*E. coli*	6 (0.9)	3 (0.6)	4 (0.8)	5 (0.8)	3 (0.5)	6 (1)
*Acinetobacter*	7 (1.1)	0 (0)	7 (1.3)	0 (0)	5 (0.8)	2 (0.3)
*N. meningitidis*	3 (0.5)	2 (0.4)	1 (0.2)	4 (0.6)	1 (0.2)	4 (0.7)
*H. influenzae*	0 (0)	1 (0.2)	0 (0)	1 (0.2)	0 (0)	1 (0.2)
*Coag. Negative*-*Staph*	0 (0)	2 (0.4)	0 (0)	2 (0.3)	1 (0.2)	1 (0.2)
*Non*-*Group*-*A*-*Strep*	2 (0.3)	0 (0)	1 (0.2)	1 (0.2)	1 (0.2)	1 (0.2)
*Salmonella*	2 (0.3)	0 (0)	0 (0)	2 (0.3)	2 (0.3)	0 (0)
Others	3 (0.5)	1 (0.2)	2 (0.4)	2 (0.3)	1 (0.2)	3 (0.5)
Total positive	34 (5.1)	22 (4.2)	27 (5.1)	29 (4.4)	23 (3.8)	33 (5.6)
Total	666 (100)	523 (100)	533 (100)	656 (100)	601 (100)	588 (100)

*CSF* cerebrospinal fluid, *LBW* low birth weight, *NBW* normal birth weight

### Year trends of bacterial meningitis cases of neonate patients

**Fig. 1:**
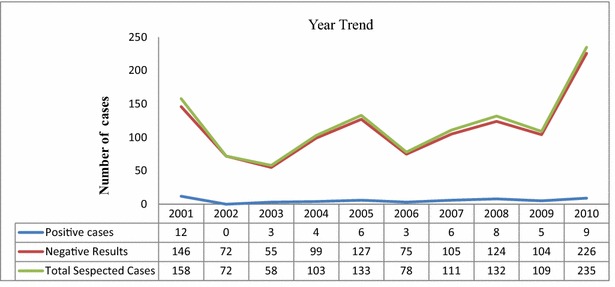
Year trends of neonatal bacterial meningitis infections isolated from CSF culture

The incidence of neonatal bacterial meningitis cases in each year revealed virtually similar isolation rate except no positive cases were identified in the year 2002, and large number of BM suspected cases have been admitted in the year 2010. Twelve (7.6%) of bacterial pathogens were isolated only in the year 2001 (Fig. 1).

## Discussion

The result of this study revealed that *S. pneumoniae* and *E. coli* were the two main etiological agents for neonatal bacterial meningitis infections at TASH. In most cases neonatal meningitis results from bacteremia (Hristeva et al. 1993; Synnott et al. 1994). In our study, the overall prevalence of bacterial meningitis infection among suspected cases of neonates in the 10 years review was (4.7%) which is higher than the previous hospital population-based retrospective study on neonatal bacterial meningitis conducted in Addis Ababa, with an overall prevalence of 1.37 per 1000 live births (Gebremariam 1998). The difference might be due to the improvement of the laboratory to increase the chance of isolation of the bacteria during appropriate culturing techniques. In contrast to our finding very high isolation rates of neonatal bacterial meningitis was reported from Kenya which was 17.9% (Laving et al. 2003), in Thailand 12.5% (Chotpitayasunondh 1994), Australia 9.2% (May et al. 2005) and Wichita 8.4% (Michael and Anne 1985).

In this study the major organisms responsible for neonatal bacterial meningitis were *S. pneumonia* 13 (23%), *E. coli* 9 (16%), *Acinetobacter* 7 (13%), *N. meningitidis* (9%), *Klebsiella* spp. 5 (9%), *S. aureus* 3 (5%), *S. pyogen* 3 (5%), *Coagulase*-*Negative*-*Staphylococcus* (4%), *Non*-*Group*-*A*-*Streptococcus* (4%), while 1 (2%) caused by *H. influenzae*. In this study the main etiological agent identified from CSF culture was *S. pneumoniae*. A large scale world health organization (WHO) study conducted in various countries of meningitis belt including Ethiopia has documented similar patterns with *S. pneumoniae* being the most common organism. It was identified in 26% of the cases. *S. pyogenes* (20%) and *E. coli* (18%) were the other common organisms (WHO 1999). *S. pneumoniae*, GBS, *E. coli* and non-typhoidal *Salmonella* species appear to predominate in East Africa (Olivia et al. 2014). However, the report from Addis Ababa revealed that the common causative organisms for neonatal meningitis were *Klebsiella pneumoniae*, *E. coli* and *Enterobacter* spp. which together accounted for 67% of all CSF isolates (Gebremariam 1998). Other studies documented that *K. pneumonia* was the common causative organisms for neonatal bacterial meningitis; Jordan 40% (Daoud et al. 1996), Iran 35.5% (Mohammad et al. 2010), Saudi Arabia (31%) (Al-Harthi et al. 2000). However, other study from Australia (Francis and Gilbert 1992), Trinidad (Ali 1995), London (Heath et al. 2003; Delouvois et al. 1991) and Canada (Stevens et al. 2003) reported that *GBS* and *E. coli* were the major etiological agents for neonatal BM infection. In developing countries, *GBS* appears to be much less frequent (Heath et al. 2003). In our study no case of *GBS* meningitis was identified; probably this organism may be rare in Ethiopia or perhaps the frequent use of ampicillin in suspected cases of neonatal infection could have stifled the isolation rate of this organism.

In our study *E. coli* was the second most common pathogen of neonatal bacterial meningitis accounted 16%. A Comparable study, Mohammad et al. (2010) in Iran reported that about 9.6% of neonatal meningitis cases were due to *E. coli*. This finding reveals that the etiological agents responsible for neonatal bacterial meningitis were slightly evenly distributed between early and late-onset meningitis, and among term and preterm newborns. In contrast to this, a study conducted in India (Ali 1995), London (Delouvois et al. 1991), Oxford (Hristeva et al. 1993) reported that *GBS* was common in early onset meningitis infections. In North America and Europe, common etiological agents for early-onset neonatal sepsis are *GBS*, *L. monocytogenes* and *E. coli* and those of late-onset sepsis include *Coagulase-Negative-Staphylococcus*, *Klebsiella* sp. and *E. coli* (Hristeva et al. 1993; Synnott et al. 1994).

In our study most cases of BM with either early- or late-onset infection occurred in LBW or VLBW newborns. Thus, as in other reports (Grupo de Hospitales Castrillo 2002; Berman and Banker 1996) birth weight appears to be an important risk factor for acquisition of BM among neonates. In our study, 29 (52%) neonates with BM had late-onset infection, whereas Chang et al. (2000) reported that from 85 patients, 51 (60%) were younger than 7 days old. Similarly, findings documented from Nigeria (Airede et al. 2008), Australia (Francis and Gilbert 1992), Trinidad (Ali 1995), Brazil (Luzia et al. 2007), Jordan (Daoud et al. 1996) and Oxford (Hristeva et al. 1993) indicated that low birth weight showed higher risk of neonatal bacterial meningitis and was significantly more likely in the preterm (p < 0.05). There is evidence to suggest that meningitis in preterm low birth weight and sickly babies is caused by organisms, usually from the maternal genital tracts (Delouvois 1994). Although advanced neonatal care enables us to save even the most preterm neonates, the very interventions sustaining those who are hospitalized concurrently expose them to serious infections due to common nosocomial pathogens (Elizabeth et al. 2013).

The highest burden of bacterial meningitis occurs in an area of sub-Saharan Africa known as the “meningitis belt”. This area is characterized by high prevalence of bacterial meningitis (Arslan 2012). Located on the eastern part of meningitis belt, Ethiopia is one of the countries which are most affected with bacterial meningitis (WHO 1999). Marked seasonal fluctuations occur in the prevalence of meningococcal meningitis which rises during the dry season from December to June with incidence as high as 1000 cases per 100,000 populations during an epidemic (Mueller and Gessner 2009). The incidence then falls steeply on the arrival of the rainy season. In this study the incidence of neonatal bacterial meningitis cases in the 10 years trend showed similar isolation rate except that no cases were identified in the year 2002.

In conclusion, we found that *S. pneumoniae* and *E. coli* were the two main etiological agents of neonatal bacterial meningitis during the 10 years review and the major causative agents for neonatal meningitis cases identified were isolated from newborns with LBW and VLBW.

This survey data had clinical input for the hospital, local health authority as well as for regional health bureau to implement appropriate meningitis surveillance against this invasive bacterial disease.
